# Acute Kidney Injury in Pregnancy: The Need for Higher Awareness. A Pragmatic Review Focused on What Could Be Improved in the Prevention and Care of Pregnancy-Related AKI, in the Year Dedicated to Women and Kidney Diseases

**DOI:** 10.3390/jcm7100318

**Published:** 2018-10-01

**Authors:** Giorgina Barbara Piccoli, Elena Zakharova, Rossella Attini, Margarita Ibarra Hernandez, Bianca Covella, Mona Alrukhaimi, Zhi-Hong Liu, Gloria Ashuntantang, Alejandra Orozco Guillen, Gianfranca Cabiddu, Philip Kam Tao Li, Gulliermo Garcia-Garcia, Adeera Levin

**Affiliations:** 1 Department of Clinical and Biological Sciences, University of Torino, 10100 Torino, Italy; 2 Néphrologie, Centre Hospitalier Le Mans, 72000 Le Mans, France; biancacovella@gmail.com; 3 Nephrology, Moscow City Hospital n.a. S.P. Botkin, 101000 Moscow, Russia; helena.zakharova@gmail.com; 4 Nephrology, Moscow State University of Medicine and Dentistry, 101000 Moscow, Russia; 5 Nephrology, Russian Medical Academy of Continuous Professional Education, 101000 Moscow, Russia; 6 Obstetrics, Department of Surgery, University of Torino, 10100 Torino, Italy; rosella.attini@gmail.com; 7 Nephrology Service, Hospital Civil de Guadalajara “Fray Antonio Alcalde”, University of Guadalajara Health Sciences Center, 44100 Guadalajara Jal, Mexico; maribaher@yahoo.es (M.I.H.); ggarcia1952@gmail.com (G.G.-G.); 8 Department of Medicine, Dubai Medical College, P.O. Box 20170, Dubai, UAE; mona_539@yahoo.co.uk; 9 National Clinical Research Center of Kidney Diseases, Jinling Hospital, Nanjing University School of Medicine, Nanjing 210000, China; zhihong--liu@hotmail.com; 10 Yaounde General Hospital & Faculty of Medicine and Biomedical Sciences, University of Yaounde I, P.O. Box 337, Yaounde, Cameroon; maglo09@hotmail.com; 11 Instituto Nacional de Perinatologia, Mexico D.F. 01020, Mexico; ale_gaba@hotmail.com; 12 Nefrologia Ospedale Botzu, 09100 Cagliari, Italy; gianfranca.cabiddu@tin.it; 13 Prince of Wales Hospital, Department of Medicine and Therapeutics, Chinese University of Hong Kong, Hong Kong; philipli@cuhk.edu.hk; 14 Department of Medicine, Division of Nephrology, University of British Columbia, Vancouver, BC V6T 1Z4, Canada; alevin@providencehealth.bc.ca

**Keywords:** pregnancy acute kidney injury (AKI), chronic kidney disease (CKD), preeclampsia, maternal death, adverse pregnancy outcomes

## Abstract

Pregnancy-related acute kidney injury (pAKI), preeclampsia (PE), and the hypertensive disorders of pregnancy are closely related conditions, which are, in turn, frequently linked to pre-existing and often non-diagnosed chronic kidney disease (CKD). The current literature and research mainly underline the effects of pregnancy complications on the offspring; this review strongly emphasizes the maternal health as well. These conditions not only negatively affect pregnancy outcomes, but have a relevant effect on the future health of affected mothers and their children. Therefore, dedicated diagnostic and follow-up programs are needed, for optimizing materno-foetal health and reducing the impact of pregnancy-related problems in the mothers and in the new generations. This narrative review, performed on the occasion of the 2018 World Kidney Day dedicated to women’s health, focuses on three aspects of the problem. Firstly, the risk of AKI in the hypertensive disorders of pregnancy (the risk is the highest in developing countries; however PE is the main cause of pregnancy related AKI worldwide). Secondly, the effect of AKI and the hypertensive disorders of pregnancy on the development of CKD in the mother and offspring: long-term risks are increased; the entity and the trajectories are still unknown. Thirdly, the role of CKD in the pathogenesis of AKI and the hypertensive disorders of pregnancy: CKD is a major risk factor and the most important element in the differential diagnosis; pregnancy is a precious occasion for early diagnosis of CKD. Higher awareness on the importance of AKI in pregnancy is needed to improve short and long term outcomes in mothers and children.

## 1. Introduction

Acute kidney injury (AKI) is still an enormous unsolved health-care problem worldwide [1,2,3]. Pregnancy-related AKI (p-AKI) is one of the most common causes of acute kidney injury in young women: it has not disappeared in high-income countries and is the leading cause of AKI in women in the developing world [1,2,3,4,5,6,7,8,9,10,11,12,13]. Even though in the past AKI was generally considered to be an all-or-nothing situation, in which complete reversal was the rule in surviving patients, this condition is now known to be associated with future risk for chronic kidney disease (CKD), hypertension, and cardiovascular diseases [14,15,16,17,18].

This narrative review, performed on the occasion of World Kidney Day 2018, dedicated to women and kidney diseases, will discuss the complex relationship between preeclampsia (PE), CKD and AKI, in order to identify fields for future intervention and research, and enable us to improve these fundamental aspects of women’s health. 

## 2. Pregnancy-Related AKI and Its Relationship with the Hypertensive Disorders of Pregnancy 

Pregnancy is a physiological situation involving the risk of death. The risk varies widely: it is modulated not only by genetic background and environmental factors, but also by country and individual income, and disempowered women in low-income countries have the highest rate of death from pregnancy-related causes, among which AKI plays a major role [13,19,20,21]. These women are not only economically disempowered, they are socially and educationally disempowered as well, leading to their inability to make informed health choices and, thus, avoid adverse outcomes.

According to Women Aid International, the probability that a pregnant African woman will die from pregnancy-related complications has been reported to be as high as 1:20, in sharp contrast with 1:2000 in the developed world. The main causes are haemorrhage, sepsis, preeclampsia-eclampsia and septic abortion, all of which also cause of AKI [13]. According to the World Health Organisation, the Maternal Mortality Ratio is 12/100,000 in developed regions and 239/100,000 in developing ones [14]. As a consequence, the pattern of p-AKI can serve as an indirect, but interesting marker of the quality of health-care delivery in pregnancy (Table 1).

The causes of p-AKI vary from one country to another, and laws on abortion and assisted fertilization are important factors as well. Septic abortion after an illegal procedure is the leading cause of early p-AKI in countries where legal abortions are not available, while PE after assisted fertilization (e.g., donation in particular) is now becoming a potential cause of p-AKI or of subsequent renal damage in developed countries [22,23,24,25,26,27,28].

As will be further discussed, the role of undiagnosed CKD, presenting as AKI in pregnancy is probably higher in developing countries, thus posing difficult clinical and ethical problems, in particular with regard to the start of chronic dialysis in pregnancy [6,29]. 

An in-depth discussion of all the causes of p-AKI is beyond the scope of this review, as it touches too many fields of medicine (nephrology, urology, infectious diseases, haematology, and immunology) and involves too many sensitive issues, such as availability and access to health care or legalisation on abortion. Many of these issues are addressed in the International Society of Nephrology’s wide-ranging “0 by 25” project, aimed at avoiding all preventable deaths due to AKI worldwide by 2025 [1,2,3]. 

**Figure 1:**
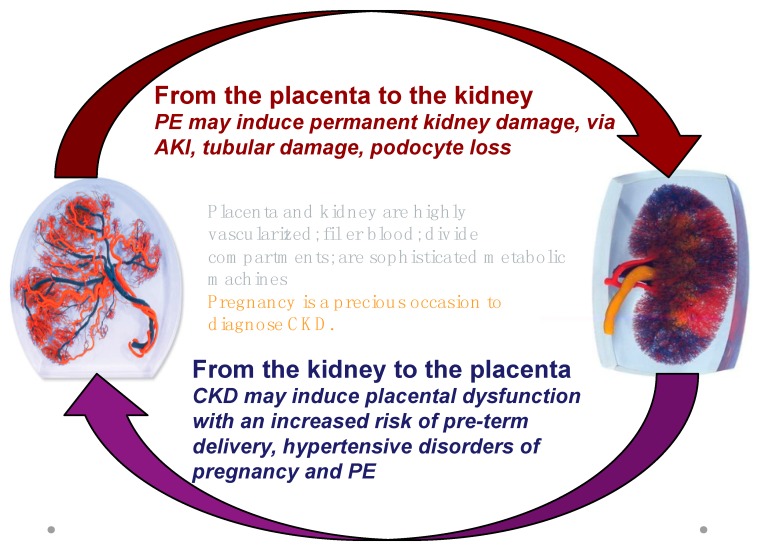
The kidney-placenta crosstalk in pregnancy (modified from the World Kidney Day 2018 editorial).

**Figure 2:**
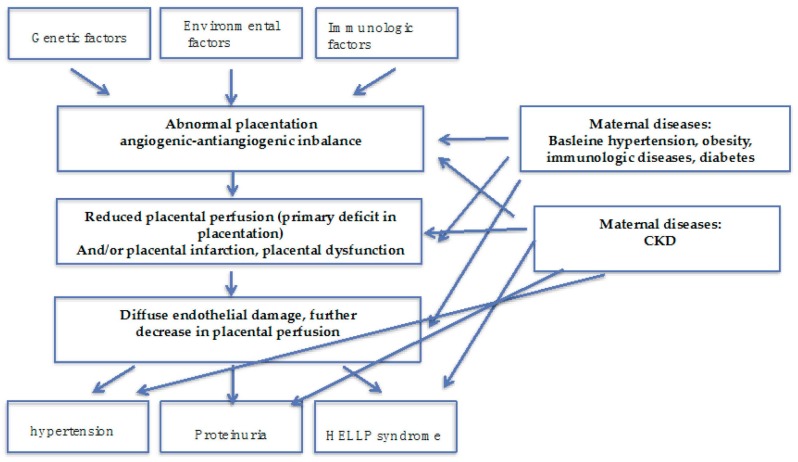
Preeclampsia is a multifaceted syndrome: some pathophysiology insights. PE may be result from a deficit in placentation, in the absence of maternal diseases; the presence of maternal diseases, mainly affecting the kidney (hypertension, diabetes, immunologic diseases) and of CKD may act at different levels modulating onset and severity of the “PE syndrome”.

Therefore, while referring to the project for all issues of general importance, we will focus on the multifaceted relationship between kidney and placenta: CKD is a risk factor for PE; most of the risk factors for PE are also risk factors for CKD (diabetes, immunologic diseases, hypertension, obesity, and the metabolic syndrome); PE is a risk factor for the development of CKD later in life (Figure 1 and Figure 2). 

The following issues will be therefore discussed: (a) The risk of AKI in the hypertensive disorders of pregnancy; (b) the effect of AKI and the hypertensive disorders of pregnancy on the development of CKD in the mother and in the offspring; (c) the role of CKD in the pathogenesis of AKI and the hypertensive disorders of pregnancy (the latter point will be further developed in a twin review on CKD and pregnancy). 

## 3. The Risk of AKI in the Hypertensive Disorders of Pregnancy 

The hypertensive disorders of pregnancy encompass a wide range of conditions of different severity, impact on kidney function and on future health [30,31,32,33,34,35]. To some extent their incidence varies according to how the conditions are defined, and to whether “superimposed” disorders are included. Overall, preeclampsia (PE) accounts for about one third of all hypertensive disorders, with a prevalence ranging from about 2%, considering only the cases developing in the absence of predisposing factors (“low-risk” pregnancy), to over 5% in unselected populations [30,31,32,33,34,35]. The modifications in the definitions over time may partially impair comparisons of historic data [30,31,32,33,34,35]. The classifications are however different, in particular for severity, as one condition (mild PE) can develop into another (severe PE); furthermore, while early PE is often severe, and is usually associated with altered biomarkers, in keeping with a primary defect of placentation, late PE may be severe and life threatening (Table 2).

Similar considerations apply to the hypertensive disorders of pregnancy, ranging from less than 10% to over 15% of pregnancies, according to whether the following disorders are included: chronic pre-conception hypertension; HELLP syndrome, the acronym for haemolysis-low platelets elevated liver enzymes, and intrauterine growth restriction (IUGR) [36,37,38,39,40]. In fact, the relationship between PE, pregnancy-induced hypertension (PIH), and HELLP is not fully clear and other conditions, such as intrauterine growth restriction, probably share a common pathogenesis [40,41,42,43,44,45]. 

**Figure 3:**
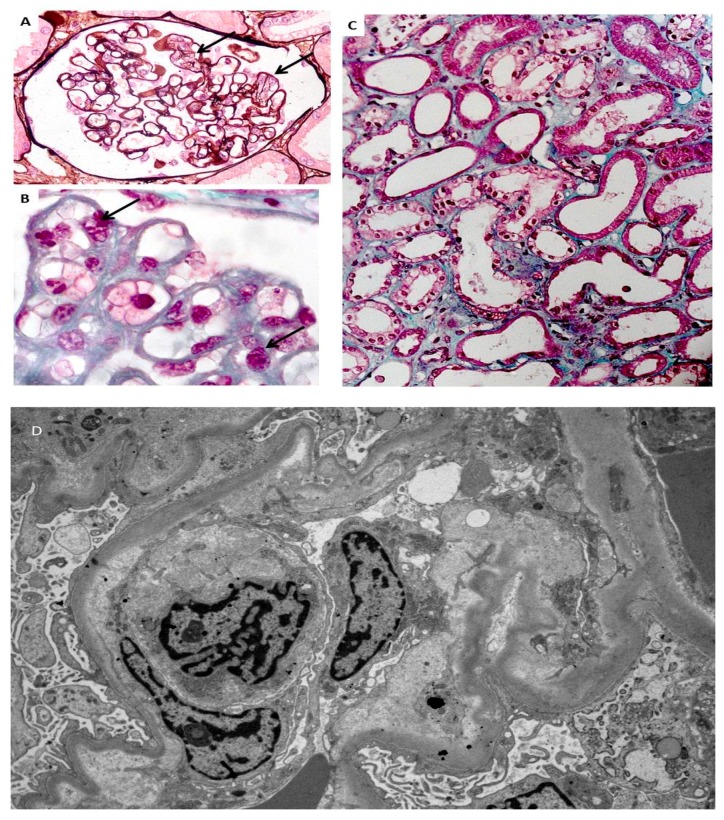
Endotheliosis is defined by endothelial changes in renal glomeruli, combining swollen endothelial cells leading to narrowed capillary lumen (**A**,**B**). Foci of acute tubular necrosis are present (**C**). Electronic microscopy shows endothelial changes (**D**) (modified from [47], with permission of the authors).

Whatever the causes, even if proteinuria, which is one of the hallmarks of PE, is usually considered as a marker of glomerular involvement, and podocyte damage is regarded as a main pathogenetic pathway, tubular damage is probably more common than previously considered [46]. In PE and all the kidney structures may be affected, ultimately resulting in p-AKI, the common pathway of other pregnancy- related noxae, such as sepsis and shock [47,48,49,50] (Table 3, Figure 3). Once more, socioeconomic status is a major component of the risk of both incidence and severity [51,52,53].

While our understanding of pathogenic mechanisms is improving, we have to acknowledge that our knowledge of the incidence of these complications of the hypertensive disorders of pregnancy is still severely limited, in particular in middle- and low-income countries, a gap which is all the more important, given the estimation of a higher incidence of both AKI and the hypertensive disorders of pregnancy in these settings [54,55]. 

## 4. The Effect of AKI and the Hypertensive Disorders of Pregnancy on the Development of CKD in Mother and Child

AKI, PE and CKD are intrinsically related, and kidney damage may represent the common link (Figure 1). A large body of evidence is accumulating on the effect of all kinds of AKI on the future development of CKD, and, in a circular spiral of risk, also affecting the hypertensive disorders of pregnancy [15,16,17,18,56,57,58,59,60].

Evidence of the long-term effect of p-AKI on the future development of CKD is limited. However, there is a growing number of studies addressed to describing the relationship between PE and the hypertensive disorders of pregnancy and the development of cardiovascular diseases and CKD [61,62,63,64,65,66,67,68,69,70,71,72,73,74,75,76,77,78,79,80,81,82,83,84,85,86,87]. The data from the literature are somewhat conflicting, as a result of the heterogeneity of the studies and the differences in the definitions they adopt. None of the available studies is prospective. Within these limits, previous PE is associated with a short-term risk of albuminuria and long-term risk of end-stage renal disease (ESRD) [64,65,66,67,68,69,70,71,72,73,74,75,76,77,78,79,80,81,82,83,84,85,86,87]. Further research on this topic is needed to make it possible to tailor long- term interventions. 

It is likely that p-AKI increases the risk of CKD, which is already higher after PE, for reasons still to be fully elucidated; as previously mentioned, as podocyte loss is a hallmark of PE, this suggests the cause is permanent glomerular damage. Endotheliosis sometimes heralds glomerulosclerosis; tubular and vascular damage may co-exist (Table 3, Figure 1, Figure 2 and Figure 3). A large recent study provides additional evidence linking AKI, PE and CKD and suggests that previous AKI is a risk factor for PE and the hypertensive disorders of pregnancy [56]. Conversely, the limit between “physiologic and pathologic” pregnancies is not fully clear, and a consistent overlap exists for pathologic findings [88,89]. For this reason, the position of the Italian Society of Nephrology (SIN), the only one so far made available by a nephrology society, is in favour of long-term follow-up of PE patients [90]; it is the only society to explicitly support this approach, based on current data. Adoption of the practice of tracking individuals longitudinally should be possible in high-income countries, especially those with electronic medical records, and this should be encouraged. In addition to maternal risks, PE is associated with intrauterine and perinatal death, pre-term delivery, and restricted intrauterine growth, the last two of which result in “small babies” (Table 4) [30,31,32].

Small babies and preterm babies have a highly increased risk of neurological deficits (a risk that is inversely proportional to gestational age), and post-natal complications, in particular those of septic origin, with further potential long-term detrimental effects [91,92,93,94,95,96,97,98,99,100,101,102]. The risks are higher in low- and mid-income countries, since survival and deficit-free survival depends to a large extent on providing intensive postnatal care [101,102,103,104,105,106,107,108].

Since kidney development is completed in the last phases of pregnancy, delayed, insufficient kidney growth, resulting in a low nephron number is the basis of the increased risk of CKD in adulthood; besides this, CKD, small, and preterm babies are at risk for the development of diabetes, metabolic syndrome, and cardiovascular diseases in adulthood [100,101,102,109,110,111,112]. The question of whether all preterm babies or only/mainly babies that are small for gestational age are at increased risk for kidney and metabolic diseases is still open, and its clarification will be useful in targeting future interventions. Interestingly, delivery of a small for gestational age baby is also a marker of risk of future CKD and cardiovascular diseases in the mother [113,114,115].

## 5. The Role of CKD in the Pathogenesis of AKI and Hypertensive Disorders of Pregnancy 

Starting in its early stages, in which it is often asymptomatic, CKD is a risk factor for PE, the hypertensive disorders of pregnancy, and preterm delivery; conversely, PE may be the first sign of undiagnosed CKD [6,90,116,117,118,119,120]. 

CKD and PE share many signs and symptoms (hypertension, proteinuria and increases in serum creatinine); while differential diagnosis may be difficult, it is not impossible during pregnancy. When signs and symptoms are correctly interpreted, and follow-up is continued after delivery, pregnancy can be a precious occasion to diagnose CKD [6,90,116,117,118,119,120,121,122,123,124]. 

The differential diagnosis can be pragmatically supported by the distinction between placental and maternal preeclampsia, the first of which is linked to a primary defect in placentation, and associated with high levels of anti-angiogenic factors and by a deep imbalance between angiogenic and anti-angiogenic factors, with impaired utero-placental flows, while the latter is a secondary effect exerted by maternal diseases, and can be expected to display a lesser and later angiogenic/non-angiogenic imbalance, together with relatively well-preserved utero-placental flows [121,123,124,125,126,127]. 

**Figure 4:**
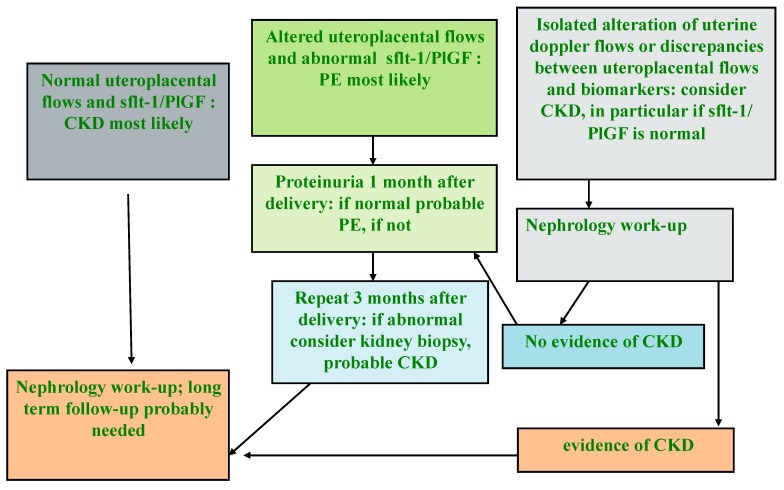
Flow chart for the differential diagnosis between CKD and PE (modified by the Authors from [90]).

It is conceivable that this Manichean differentiation does not capture all the important issues in the pathogenesis of PE, and that a “mixed” pathogenesis may frequently be present. However, the analysis of circulating biomarkers, namely Soluble fms-like tyrosine kinase- (sFlt-1) and placental growth factor (PlGF), the two that have most often been studied in detail), and of utero-placental flows, where available (Doppler study is economically less demanding, and can be performed rapidly at the bedside) can support the differential diagnosis between CKD and PE and help guide post-pregnancy follow-up (Figure 4) [123,124,125,126,127]. 

Moreover, because making a differential diagnosis between acute damage and chronic kidney disease (AKI vs CKD) may be difficult during pregnancy, it is important to carry out extensive kidney evaluation after delivery, once the physiological changes related to pregnancy have been resolved (Figure 4).

Unfortunately, these intentions may be impossible to put into practice in situations where they are in contrast with the harsh reality of insufficient care and inadequate attention to the early diagnosis of CKD. Long-term studies are needed to establish the link between the different clinical and subtle-subclinical changes observed in formerly preeclamptic women, as an ongoing investment in health care for the young.

## 6. The Role of Immunologic Diseases in the Pathogenesis of the Hypertensive Disorders of Pregnancy and p-AKI

Among the immunologic diseases, systemic lupus erythematosus (SLE) principally affects women of childbearing age, and is the most recognized risk factor for the hypertensive disorders of pregnancy and p-AKI [128,129,130,131]. 

The most common feature of kidney damage in SLE is lupus nephritis (LN), characterised by glomerular involvement, but interstitial and vascular lesions also contribute to CKD. Patients affected by SLE share with other CKD patients the traditional risk factors for adverse pregnancy outcomes, but they have also specific risks of p-AKI including lupus flares, preeclampsia, HELLP syndrome, and thrombotic events. The major predictors for acute pregnancy-related complications are Class III and IV lupus nephritis, a previous history of renal flares, longer disease, hypocomplementemia, antiphospholipid syndrome (APS), and the presence of antiphospholipid antibodies [132,133,134,135,136]. In this context, high titres and triple positivity for antiphospholipid antibodies, previous thrombosis and the presence of a lupus anticoagulant are associated with a higher risk of severe maternal and foetal complications, both in primary and secondary APS, and in catastrophic APS (CAPS) the most severe form of the syndrome [136,137,138,139,140,141,142]. Conversely, the occurrence of the HELLP syndrome in a patient with APS should raise the suspicion that CAPS will shortly be manifested [139,141,142,143].

In addition to CAPS, thrombotic thrombocytopenic purpura and atypical haemolytic-uremic syndrome comprise the continuum of pregnancy-related thrombotic microangiopathies (TMA), associated with high maternal and foetal morbidity and mortality, including p-AKI [139,144,145,146,147]. Pregnancy, itself a procoagulant state, is a trigger for thrombotic thrombocytopenic purpura, especially in the setting of *ADAMTS-13* deficiency. A deficiency of *ADAMTS-13* is responsible for most cases of thrombotic thrombocytopenic purpura, generally occurring in the second and third trimester of pregnancy. Pregnancy also induces complement activation and is a trigger for atypical haemolytic uremic syndrome, associated with complement dysregulation, which mainly occurs post partum [139,144,145,146,147].

After delivery, inflammation, the release of foetal cells in the maternal circulation, infections, and haemorrhage can lead to activation of the alternative complement pathway, which, in the absence of effective regulatory mechanisms, may induce postpartum atypical haemolytic-uremic syndrome (aHUS); complement dysregulation was also found to be associated with the HELLP syndrome, which shares several features with pregnancy-associated TMA [148,149,150,151,152]. The new frontiers of treatment with eculizumab make diagnosis of pivotal importance for allowing timely treatment [149,150,151,153].

## 7. Conclusions: The Need for Research and Clinical Intervention

Research is increasingly being done on the short- and long-term effects of p-AKI and the hypertensive disorders of pregnancy on the health of mothers and children, and studies are generally in agreement in highlighting an increased risk for both [60,61,62,63,64,65,66,67,68,69,70,71,72,73,74,75,76,77,78,79,80,81,82,83,84,85,86,87]. However, study designs and definitions are heterogeneous and there is need for establishing and validating a common lexicon in this regard, to allow integration of the available data and, more importantly, make possible a detailed, comparable set of information for the future studies. 

While information on the short-term effects of p-AKI and the hypertensive disorders of pregnancy is available in differently resourced settings, data on the long-term effects are limited to highly-resourced countries, and even then tend to be sparse and non-uniform, thus underlining the need for more research in the fragile populations that are most susceptible both to p-AKI and other kidney diseases. 

While the new generation of biomarkers of preeclampsia and related disorders facilitates better understanding of the pathogenesis and prognosis of the hypertensive disorders of pregnancy, there is a need for reliable, simple and inexpensive prognostic markers of the effects of p-AKI and the hypertensive disorders of pregnancy on future health. Clinical care of p-AKI, including dialysis care, should be a priority, as maternal health is fundamental not only for the patient, but also for her family and society [1,2,3].

Treatment for p-AKI and the hypertensive disorders of pregnancy should not be discontinued at delivery or shortly afterwards, and long-term programs should be established at least for women with evidence of chronic kidney disease. Children born to p-AKI mothers, born pre-term or small for gestational age should be followed up to optimize prognosis. All these are ambitious tasks. It will be up to the nephrology community to ensure that they are not seen as being overambitious.

## Figures and Tables

**Table 1:** A general, but non-exhaustive classification of the main causes of pregnancy-related AKI, in the early and late phases of pregnancy.

Main Clinical Feature	Phase	Condition	Main Clinical Features
**Prerenal**			
Hypovolemic	Early	Hyperemesis gravidarum	May be severe, associated with severe nutritional deficits, so more common in patients with a nutritional disorder or malnutrition; may reflect psychological problems. More commonly diagnosed in developed countries, it is probably underestimated in developing ones.
	Any time	Other causes of hypovolemia	Infectious diseases, nutritional disorders; acute fatty liver of pregnancy; metabolic acidosis, uremia.
Haemorrhagic	Early	Abortion	Early foetal loss can cause severe haemorrhaging, but unsafe, illegal abortions are the most common cause of massive bleeding, usually associated with sepsis. More common in low- and middle-income countries, and where abortion is illegal.
	Late	Placental abruption	Can cause massive bleeding, as well as foetal loss, usually occurs in late pregnancy.
Hypotensive	Any time	Hypovolemia, cardiopathy, sepsis	Hypotension is usually a concomitant cause and a marker of severity of the above. The rare, but sometimes severe, cardiomyopathy of pregnancy can lead to severe hypotension and AKI. Sepsis (any cause, any phase) is often associated with hypotension up to hypotensive shock, and associated with tubular necrosis (see below).
Combined pathogenesis	Any time	Septic abortion, placental abruption, puerperal sepsis	Severe bleeding is associated with hypovolemia and hypotension. While the “usual” classification of AKI may be of help, focusing on one element only may avert attention from treating all associated factors.
**Parenchymal (for PE, and related disorders, see further tables)**			
Glomerular	Any time	CKD (known or undiagnosed)	The presence of CKD is associated with adverse pregnancy outcomes starting from the early stages. Immunologic diseases may relapse or appear in pregnancy. CKD worsening is described in 20–80% of patients.
	Usually late	Microangiopaties	Haemolytic uremic syndrome and related diseases are an emerging concern in particular in developed countries probably because they fail to be diagnosed in low-income settings.
Interstitial damage	Any time	Iatrogenic, associated with other causes of AKI	The causes are the same encountered outside of pregnancy but the consequences may be more severe. Whether the “pregnant kidney” is associated with increased risk is a matter of debate. History of obstructive renal disease and vesicoureteral reflux may contribute to loss of parenchymal volume which for still unknown reasons may be facilitate PE and possibly AKI
	Any time	Pyelonephritis and upper urinary tract infections	These infections seldom cause AKI, even though they can be severe and life threatening. In this context, AKI is usually linked to sepsis or is iatrogenic.
Combined pathogenesis	Any time	Tubular necrosis- cortical necrosis	Tubular necrosis may result from any severe AKI, and may be multifactorial.
**Postrenal-Obstructive**			
Obstruction of the urinary tract	Any time	Stone disease	Hypercalciuria can occur in pregnancy. Pain due to the passage of a stone may be misinterpreted, especially at term; infection and undiagnosed obstruction may be life- and function-threatening.
	Post partum	Iatrogenic	Ligature of the ureters is a rare but serious iatrogenic complication of caesarean section or reinterventions.
	Any time	Neoplasia	Kidney and urinary tract neoplasia are rare in young women. Diagnosis should be considered in macroscopic haematuria, in particular if there are clots.
	Usually late	ADPKD and other cystic diseases of the kidney	Cystic diseases of the kidney may go unrecognized in younger women. Large non-symptomatic cysts may become symptomatic, cause pain or obstruction, or become infected in pregnancy.
Functional	Usually late	Functional obstruction and hydronephrosis	Mild urinary tract dilatation (usually on the right side) is common, and usually without consequences; pyelo-ureteral junction anomaly, may decompensate, leading to giant hydronephrosis. Severe urinary tract dilatation is described in patients with reflux nephropathy.
Combined pathogenesis	Any time	Infection, bleeding in obstructed kidney	Different causes may be associated; when sepsis (mainly Gram negative), is superimposed, AKI may be of combined parenchymal (septic), prerenal (hypovolemia, shock) and obstructive pathogenesis.

**Table 2:** Preeclampsia: focus on classifications and definitions.

Term	Definition	Main Problems and Limits
Preeclampsia (PE)	Hypertension and proteinuria or end-organ damage in a previously healthy woman; involvement is reversible 1–3 months after delivery	Usually defined as new onset of proteinuria above 300 mg/day and hypertension after the 20th gestational week (GW) in a patient who was previously normotensive and without proteinuria or kidney disease. The syndrome resolves within three months from delivery. New definitions include hypertension in the absence of proteinuria but in the presence of end-organ damage, including creatinine increase. Does not apply to patients with chronic kidney disease or on dialysis, due to baseline hypertension, proteinuria, or to no urine output.
Eclampsia	Same as above, with neurological damage and convulsions	This definition is no longer universally accepted. Some authors consider it synonymous with untreated (or inadequately treated) PE.
Mild PE	PA ≥140/90 <160/110 proteinuria ≥0.3 <5.0 g/day in the absence of the criteria set forth above	The definition of mild and severe PE is somewhat static; mild PE can abruptly evolve into severe PE. Therefore, this definition should be used to: identify ALL cases of severe PE, that should be followed accordingly; identify cases in which the disease is not severe, allowing pregnancy to continue under careful surveillance, keeping in mind that mild PE can abruptly progress to severe PE.
Severe PE	Central nervous system involvement Liver damage Poorly controlled hypertension Proteinuria ≥5 g/24 h Platelets <100.000 Oliguria <500 mL/24 h Pulmonary oedema Intrauterine growth restriction (IUGR)	
Early PE	Before 34 completed GW	This pragmatic definition has the advantage of simplicity, and makes the early identification of cases possible; however a late diagnosis of early PE may in fact be identified as “late” PE.
Late PE	After 34 completed GW	
Maternal PE	With maternal predisposing disease	This pragmatic definition is clinically relevant (maternal PE is usually, but not uniformly milder); the definition of maternal disease is elusive, and the equation early = placental = severe; late = maternal = mild is imperfect and may be misleading.
Placental PE	Severe placental involvement in the absence of the above	
Angiogenic PE	Altered angiogenic/non-angiogenic balance	This is a promising approach, with the advantage of simplicity and of employing numeric values, and is possibly more objective; however, there is no agreement on how best to test for the condition: cut-points are not univocal, the levels and the ratio between factors may change over time and the availability of the tests is limited.
Non angiogenic PE	Absence of the above	
Superimposed PE	PE with underlying CKD	Some but not all CKD are proteinuric or hypertensive before pregnancy; this definition tries to correct for the baseline values. However, there is no agreed level of “worsening” of proteinuria or hypertension, in part because of the adaptation of anti-hypertensive treatments in pregnancy. Albeit of potential interest, this definition is ambiguous and less frequently encountered.
Postpartum PE	A clinical syndrome with the clinical features of PE, occurring postpartum	A rare occurrence, probably accounting for less than 5% of PE. The clinical picture is often severe and onset is abrupt; diagnosis may be difficult and delayed, since it may occur after hospital discharge.
**Other related definitions (other hypertensive disorders of pregnancy)**		
Pregnancy induced hypertension (PIH)	“Isolated” hypertension in pregnancy	Hypertension occurring after the 20th week of pregnancy in a previously normotensive woman; the definition is the same as for PE; the absence of proteinuria is however no longer sufficient to exclude PE (see above).
Pregnancy induced proteinuria	“Isolated” proteinuria in pregnancy.	Proteinuria above 300 mg/day occurring after the 20th week of pregnancy in a previously non-proteinuric woman. Often a sign of underlying CKD.
HELLP syndrome	A virulent syndrome of endothelial damage	The acronym stands for hypertension, elevated liver enzymes, low platelets (HELLP); it is a severe syndrome, often abrupt, potentially occurring in the immediate postpartum period. Some authors consider it as the end of the spectrum of PE (severest disease); others consider it a separate disease, due to the frequent lack of prodromal PE.
IUGR	Intrauterine growth restriction	Some authors relate IUGR to the hypertensive disorders of pregnancy, due to its pathogenesis, which is related to insufficient placental vascularization. See Table 3 for details.

**Table 3:** Kidney involvement in pregnancy: pathologic findings.

Structure	Main Clinical Feature	Pathologic Findings	Long-Term Effects
Glomerular	Isolated proteinuria, PE, AKI	Glomerular endotheliosis (PE), focal-segmental glomerulosclerosis (FSGS); podocyte loss. Differential diagnosis with CKD	Endotheliosis is the hallmark of PE. It is considered reversible, and may also be found in normal pregnancies. FSGS is not reversible. Its frequency has been differently assessed, and the symptoms of severe endotheliosis may merge with it. Podocyte loss, with permanent damage to the basal membrane seems to be the unifying cellular lesion.
Tubular	AKI, severe PE, HELLP	Acute tubular necrosis, tubulitis, no alteration	AKI can be an outcome of ischemic, hypovolemic. septic or toxic (iatrogenic) damage. The long-term effects of AKI are not fully understood, but severe dialysis-requiring AKI even when reversible in the short term, is associated with CKD and ESRD later in life.
Vascular	Hypertensive disorders, PE, AKI	Vascular hypertrophy, up to “onion skin” lesions; ischemic or necrotic glomerular changes	Hypertensive crises in pregnancy may exhibit the features of “malignant” or accelerated hypertension. While onion-skin changes indicate a progressive onset, necrotic changes are the hallmark of the abrupt development of severe hypertension.
Global	AKI followed by CKD	Cortical necrosis	The irreversible loss of function associated with necrotic changes is often multifactorial (hypovolemic or haemorrhagic shock, sepsis, HELLP); can be diagnosed using magnetic resonance imaging.

**Table 4:** “Small babies”: focus on classifications and definitions.

Term	Definition	Main Problems and Limits
Small baby	A baby weighing less than 2500 g at birth	While there is a trend towards an inverse association between birth weight and clinical problems, the outcome of “small babies” depends on the pathogenesis of low birth weight; hence, measures adjusted for gestational age and growth patterns show a better relationship with short- and long-term outcomes.
Very small baby	A baby weighing less than 1500 g at birth	
Pre-term delivery	Delivery before 37 completed gestational weeks; late pre-term: 34–37 gestational weeks	These three widely-used terms were defined in relation to “normal development” (at term); higher risk for mild developmental or intellectual deficits (late pre-term is mainly associated with school problems); extreme preterm is associated with higher frequency of mild deficits, and higher incidence of severe developmental neurologic problems. The risk for metabolic syndrome, hypertension and CKD, albeit less well explored, may follow a similar pattern. Once more, these definitions should be associated with data on intrauterine growth; outcome may be modified by follow-up.
Early pre-term delivery	Delivery before 34 completed gestational weeks	
Very early (or extreme) pre-term delivery	Delivery before 28 completed gestational weeks	
Small for gestational age baby (SGA)	A baby below the 5th or the 10th centile for gestational age, adjusted to local growth curves	This finer definition takes into account the relationship between being small or preterm and the “quality” of intrauterine growth. This definition does not distinguish between harmonic growth of genetically small children (not pathologic) and stunted growth of children not attaining their genetic growth target. Given the wide ethnic differences in growth curves, the absence of local data may impair precise interpretation. Other relevant terms are AGA (adequate for gestational age) and LGA (large for gestational age: above the 90th or the 95th centile)
Intrauterine growth restriction (IUGR)	A baby that does not reach its growth potential, i.e. one below the 5th centile for gestational age, or whose growth curve flattens in pregnancy	Growth restriction is a dynamic concept that indicates flattening of the growth curve. Data are emerging on the pivotal importance of the “quality” of intrauterine growth, which may be more important than actual birth weight, or prematurity; while babies who are SGA below the 5th centile are so often IUGR that they are included in the definitions, a baby may still be adequate for gestational age, be born at term, but be IUGR, if its growth was severely reduced in the last gestational weeks.
